# Cerebral Vasospasm with Ischemia following a Spontaneous Spinal Subarachnoid Hemorrhage

**DOI:** 10.1155/2013/934143

**Published:** 2013-02-14

**Authors:** Sophia F. Shakur, Hamad I. Farhat

**Affiliations:** ^1^Section of Neurosurgery, The University of Chicago Medicine, Chicago, IL 60637, USA; ^2^Department of Neurosurgery, NorthShore University HealthSystem, 2650 Ridge Avenue, Evanston, IL 60201, USA

## Abstract

Cerebral vasospasm is a well-known consequence of aneurysmal subarachnoid hemorrhage (SAH) triggered by blood breakdown products. Here, we present the first case of cerebral vasospasm with ischemia following a spontaneous spinal SAH. A 67-year-old woman, who was on Coumadin for atrial fibrillation, presented with chest pain radiating to the back accompanied by headache and leg paresthesias. The international normalized ratio (INR) was 4.5. Ten hours after presentation, she developed loss of movement in both legs and lack of sensation below the umbilicus. Spine MRI showed intradural hemorrhage. Her coagulopathy was reversed, and she underwent T2 to T12 laminectomies. A large subarachnoid hematoma was evacuated. Given her complaint of headache preoperatively and the intraoperative finding of spinal SAH, a head CT was done postoperatively that displayed SAH in peripheral sulci. On postoperative day 5, she became obtunded. Brain MRI demonstrated focal restricted diffusion in the left frontoparietal area. Formal angiography revealed vasospasm in anterior cerebral arteries bilaterally and right middle cerebral artery. Vasospasm was treated, and she returned to baseline within 48 hours. Spontaneous spinal SAH can result in the same sequelae typically associated with aneurysmal SAH, and the clinician must have a degree of suspicion in such patients. The pathophysiological mechanisms underlying cerebral vasospasm may explain this unique case.

## 1. Introduction

Aneurysmal subarachnoid hemorrhage (SAH) accounts for only 1%–7% of all strokes but is responsible for 27% of all stroke-related years of life lost before the age of 65 years [1]. Delayed cerebral ischemia (DCI) is a common complication of aneurysmal SAH and is associated with poor clinical outcome and death [2]. Cerebral vasospasm—arterial narrowing occurring 3 to 14 days after aneurysmal SAH—is considered the main culprit of DCI [3, 4]. Numerous studies subsequently performed on the pathophysiology of cerebral vasospasm revealed a complex cascade of events, triggered by blood breakdown products of the subarachnoid blood clot, that include disrupted function of the physiologically vasoactive molecules of nitric oxide (NO) and endothelin-1 (ET-1) as well as inflammation [5–8]. Although the recent CONSCIOUS-1 clinical trial failed to show significant reductions in DCI or improved outcomes despite the decreased rates of vasospasm, suggesting that vasospasm plays a smaller role in the pathogenesis of DCI than previously thought, Crowley et al. demonstrated a strong correlation between angiographic vasospasm and cerebral infarction using data from the CONSCIOUS-1 database [9–11]. Additionally, in the CONSCIOUS-3 study, higher doses of Clazosentan significantly reduced post-SAH vasospasm-related morbidity [12]. 

Here, we present the first case of cerebral vasospasm with ischemia following a spontaneous spinal SAH. We assert that our case reaffirms the paradigm of cerebral vasospasm as the primary cause of DCI after SAH. Furthermore, we believe that blood breakdown products reached the cerebral vasculature through cerebrospinal fluid (CSF).

## 2. Case Report

### 2.1. History and Examination

**Figure 1:**
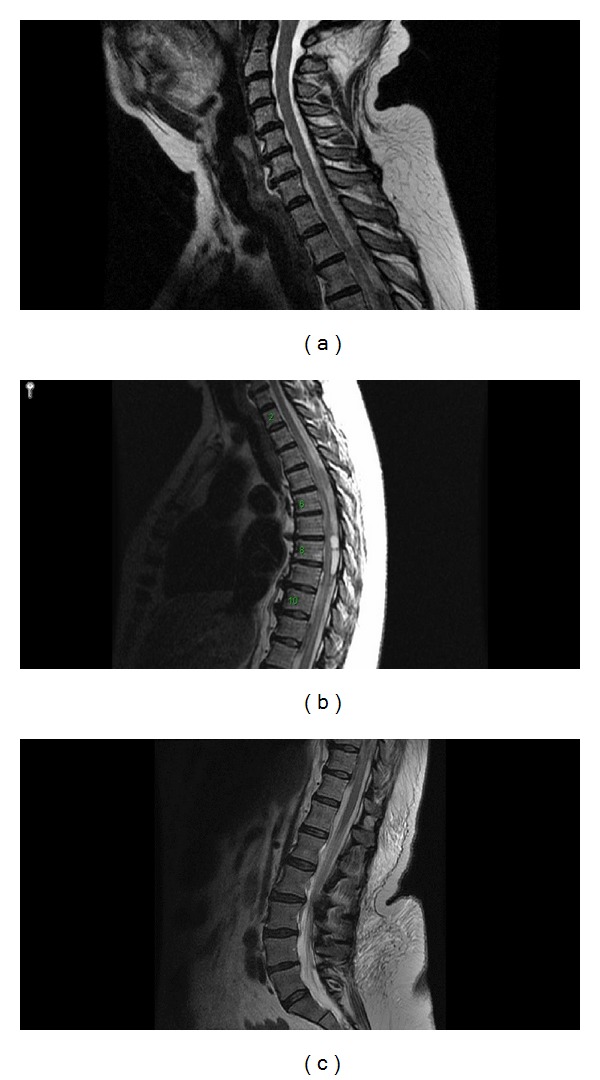
Sagittal T2 MR images of the cervical (a), thoracic (b), and lumbar (c) spine showing a complex cystic lesion in the dorsal spinal canal, spanning from above T1 to below T11, with spinal cord compression. Additionally, there is mildly increased T2 signal centrally within the spinal cord from the T3 to T11 level.

This 67-year-old woman, who was on Coumadin for atrial fibrillation, presented to an outside hospital emergency room on January 9, 2011, with sudden onset chest pain that radiated to the back and was associated with headache as well as leg paresthesias. Cardiac workup, including cardiac enzymes, electrocardiogram, echocardiogram, chest X-ray, and computed tomography (CT) aortic dissection protocol, showed no evidence of acute myocardial infarction, aortic dissection, or pulmonary embolism. Of note, the patient had an international normalized ratio (INR) of 4.5 and a prothrombin time (PT) of 40.2, and so her Coumadin was held. Approximately 10 hours after presenting to the outside hospital, the patient complained of bilateral leg weakness and numbness. On examination, she had complete loss of movement in both legs and lack of sensation below the umbilicus. She also had loss of volitional rectal tone. Magnetic resonance (MR) imaging of the spine with and without contrast was obtained and showed a complex cystic lesion in the dorsal spinal canal, spanning from above T1 to below T11, with spinal cord compression (Figure 1). This radiographic finding was the most suspicious for an intradural hemorrhage. Consequently, the patient was transferred to our institution for emergent neurosurgical intervention. She was given recombinant factor VIIa, fresh frozen plasma, and vitamin K for reversal of anticoagulation and was taken to the operating room. Despite the fact that the patient's presentation included headache with an INR of 4.5, a preoperative head CT scan was not obtained, since there was no report of a thunderclap-like headache and the patient's neurological deficits localized the lesion to the spinal cord.

### 2.2. Operation

**Figure 2:**
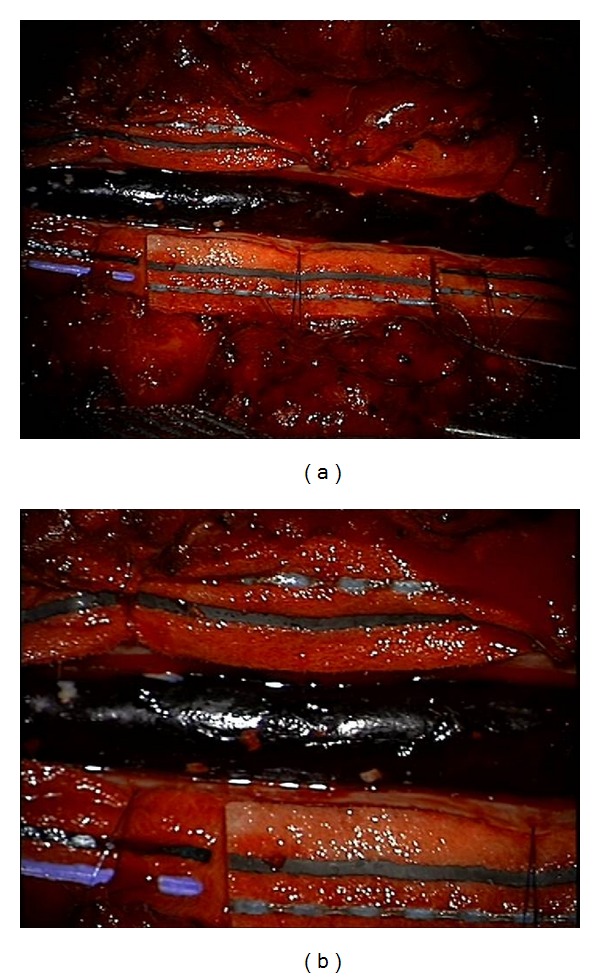
Intraoperative photographs taken using the operating microscope displaying (a) a large blood clot under the subarachnoid layer overlying the spinal cord and (b) arachnoid granulations on the surface of the hematoma.

The patient emergently underwent T2 to T12 laminectomies. No epidural hematoma was found. We then performed a durotomy from T2 to T12. A large intradural blood clot was encountered. Using the operating microscope, the hematoma was seen to be in the subarachnoid space (Figure 2). The arachnoid membrane was opened and the hematoma was evacuated. Although a large vein was identified inferiorly, it did not appear to be arterialized and there was no evidence of a vascular malformation. An intraoperative specimen of the hematoma was sent to pathology. Final histopathology was consistent with thrombus. 

### 2.3. Postoperative Course

**Figure 3:**
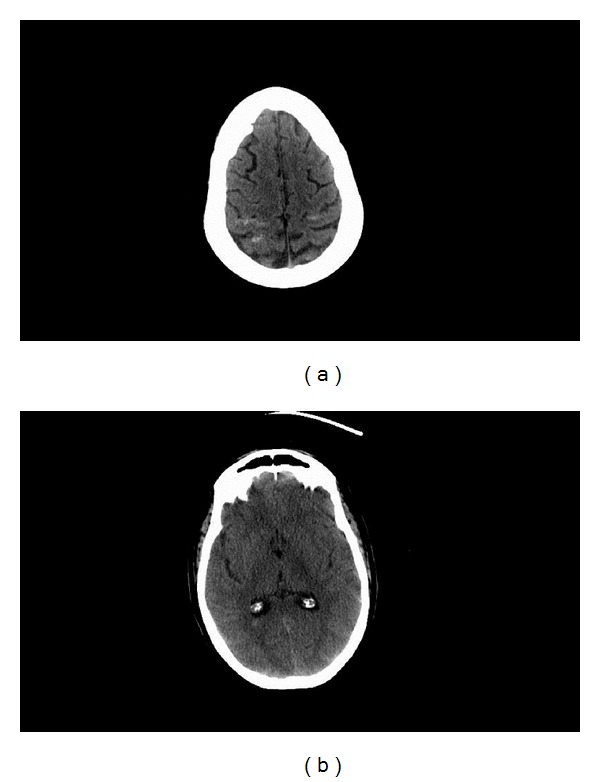
Axial CT head images without contrast on postoperative day 0 demonstrating subarachnoid hemorrhage bilaterally in the posterior frontoparietal regions (a) and hemorrhage in the occipital horn of the right lateral ventricle (b).

**Figure 4:**
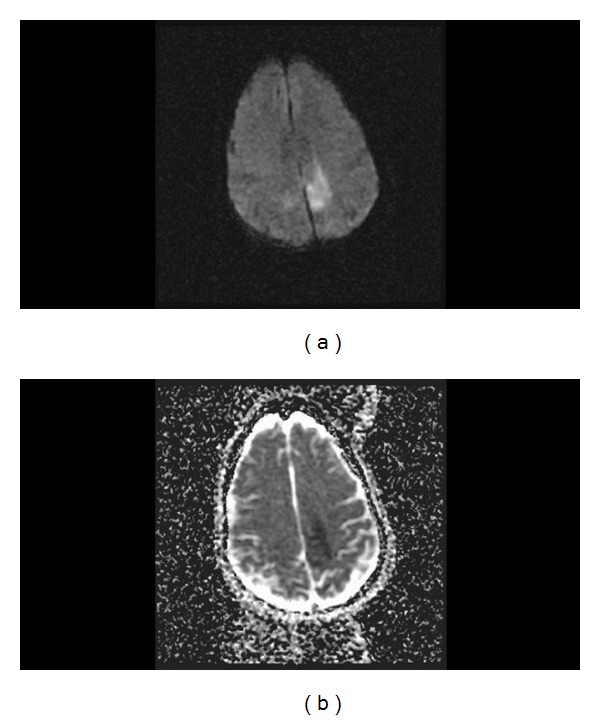
Axial diffusion-weighted (a) and ADC (b) MR images of the brain on postoperative day 5 revealing focal restricted diffusion and low ADC signal in the left posterior frontoparietal area consistent with acute infarction.

**Figure 5:**
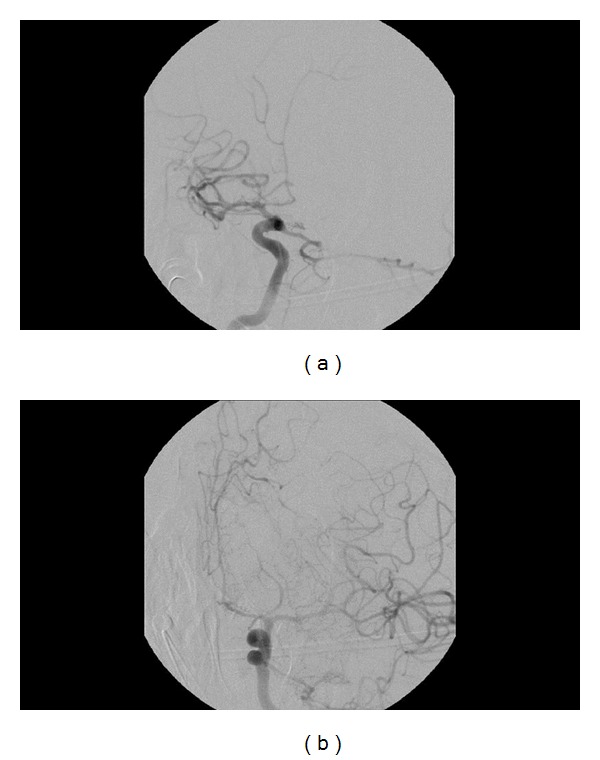
Formal cerebral angiogram performed on postoperative day 5. (a) Right ICA injection demonstrating vasospasm in the right ACA and the right frontal M2 branch with no flow restriction. (b) Left ICA injection showing vasospasm in the left ACA.

The patient was admitted to the intensive care unit (ICU) for postoperative observation. She remained in full strength in bilateral upper extremities but without movement and sensation in bilateral lower extremities. Given the patient's complaint of headache preoperatively and the intraoperative finding of a spinal subarachnoid hemorrhage, a head CT without contrast was done postoperatively (Figure 3). It showed some subarachnoid hemorrhage bilaterally in the posterior frontoparietal regions and hemorrhage in the occipital horn of the right lateral ventricle. No blood was identified in the basal cisterns or Sylvian fissures bilaterally. A head CT angiogram was subsequently obtained, which revealed no aneurysm or vascular malformation and no vasospasm. Plans were made to perform a diagnostic spinal angiogram. On postoperative day 5, however, the patient became obtunded. On examination, pupils were equal and reactive to light and she opened her eyes to painful stimulation, briskly localized to pain with her left arm, and withdrew to pain with her right arm. She was hemodynamically stable, and there was no witnessed seizure activity. A head CT head without contrast showed no significant interval changes. MR imaging and angiography of the brain was then done, demonstrating focal restricted diffusion in the left posterior frontoparietal area consistent with acute infarction (Figure 4). Ischemic stroke work-up included a transthoracic echocardiogram that showed no left atrial thrombus. Other possible etiologies that could account for the concomitant encephalopathy were vasospasm and subclinical seizures. Since the patient was afebrile and had no leukocytosis, meningitis was not investigated as a likely cause. Electroencephalography (EEG) did not show any epileptiform discharges. Formal cerebral and spinal angiograms performed on postoperative day 5 revealed vasospasm in the anterior cerebral arteries bilaterally as well as vasospasm in the right middle cerebral artery (Figure 5). On the spinal angiogram, there were no dural arteriovenous fistulas, arteriovenous malformations, or other vascular pathologies appreciated. The cerebral vasospasm was treated aggressively with medical management, and she was monitored clinically and found to improve back to her baseline within 48 hours. However, on postoperative day 20, her mental status declined abruptly. A head CT head without contrast was stable. Medical work-up included abdomen and pelvis CT without contrast showing a large amount of pneumoperitoneum consistent with bowel perforation. The patient expired on the following day, and no autopsy was performed to further determine the cause of death. 

## 3. Discussion

Cerebral vasospasm is a type of arterial vasoconstriction characterized by a prolonged, often severe, but reversible intracranial arterial narrowing that occurs 3 to 14 days after an aneurysmal SAH [3, 4, 13]. Angiographic vasospasm was first described by the neurosurgeon Ecker and the radiologist Riemenschneider in 1951 [13, 14]. They astutely recognized that cerebral vasospasm only occurred after a delayed period following the hemorrhage was the maximal closest to the aneurysm and was the main cause of mortality and morbidity in patients with aneurysmal SAH who did not have clear evidence of aneurysm rebleeding.

In 1980, Fisher et al. established the importance of the thickness and the location of the subarachnoid blood clot in the development of cerebral vasospasm [15]. More specifically, they demonstrated a relationship between the SAH seen on CT soon after the ictus and the risk for vasospasm. They also provided a grading scale based on the thickness and the location of the blood—particularly in the basal cisterns—that was predictive of cerebral vasospasm. Although this scale has been criticized, studies have repeatedly shown that the risk of symptomatic vasospasm is directly related to the amount of initial cisternal SAH [16, 17]. These findings have been corroborated further by the absence of vasospasm in cases of nonaneurysmal SAH. For example, thick, cisternal SAH is found in less than 10% of patients with closed head injury, and so post-traumatic vasospasm is rarely seen and the benefit of monitoring these patients for vasospasm has not been proven [13]. Additionally, vasospasm is an uncommon complication of arteriovenous malformation (AVM) rupture, which has been justified by the lack of large volume SAH from AVMs [13, 18]. Finally, the small quantity of blood in the prepontine cistern observed with spontaneous perimesencephalic SAH is associated with a benign natural history, including a low risk for vasospasm [13, 19].

Cerebral vasospasm, then, is not a general consequence of SAH, but rather it is the endpoint of a specific pathophysiological cascade that typically begins with aneurysmal SAH. Indeed, over the past several decades, extensive research has been conducted on the pathogenesis of vasospasm. These investigations identify oxyhemoglobin (OxyHb) as the main mediator of cerebral vasospasm [6, 20, 21]. Next, we further describe the role of OxyHb, reviewing the properties of CSF after SAH as well as the mechanisms of OxyHb-induced vasoconstriction.

Histopathology studies reveal that within 24 hours after SAH, there is an intense polymorphonuclear cell infiltration [6]. Phagocytosis and breakdown of red blood cells begin within 16 to 32 hours, peaks around day 7—coinciding with the peak vasospasm period after SAH—and continues for several days. After day 7, the inflammatory response quells, and by day 10, fibrosis ensues. Phagocytosis and breakdown of red blood cells in CSF results in the appearance of OxyHb 2 hours after SAH and the presence of bilirubin by day 4. Over the course of the next week, the level of OxyHb decreases and that of bilirubin increases. Ultimately, the pathology of the subarachnoid space and the analysis of xanthochromic CSF show that OxyHb is present during peak vasospasm. Confirmation of the correlation between CSF OxyHb concentration and vasospasm, though, has been difficult because lumbar CSF OxyHb levels do not accurately reflect concentrations adjacent to spastic arteries [22, 23]. Nonetheless, two studies demonstrated that vasospasm is dependent on periarterial OxyHb concentration [21, 22].

Interestingly, in our case, there was only a small amount of intracranial subarachnoid blood in the peripheral sulci with no blood in the basal cisterns, but the patient developed cerebral vasospasm with ischemia. We reconcile this discrepancy by suggesting that the hemorrhage load located in the spinal subarachnoid space produced molecules carried in the CSF that initiated a pathophysiological cascade, as described previously, and resulted in cerebral vasospasm. This case, then, substantiates our current understanding of the putative mediators in the pathogenesis of vasospasm, namely, initial blood load and released factors. Moreover, our case provides evidence that the vasoactivity of xanthochromic CSF is due to OxyHb. 

 In their review of spontaneous spinal SAH, Domenicucci et al. identified a total of 69 cases [24]. The most common etiologies identified were coagulopathy (40.5%), lumbar puncture (44.9%), and traumatic injury (15.9%). Radiological diagnosis was difficult, since MR imaging and CT could not differentiate between subarachnoid and subdural lesions, and so most cases were diagnosed on the basis of surgical or autopsy findings. Overall mortality was 25.7%. Outcome of treatment—usually surgery—was good in 93.5% of patients with satisfactory neurological status on presentation and 15.8% of patients with severe neurological deficits. None of these cases of spontaneous spinal SAH were noted to have concomitant intracranial SAH.

 Our review of the literature, however, revealed 2 cases of spontaneous spinal SAH associated with cerebral SAH [25, 26]. One case was recently documented in the Spanish literature, and the other evolved from a complicated lumbar puncture. We posit that the number of such cases is probably underestimated, since the brain is not routinely imaged in spinal SAH cases. Our review also identified no cases of spontaneous spinal SAH associated with global neurological injury. Thus, to our knowledge, we report the first case of cerebral vasospasm with ischemia following a spontaneous spinal SAH. Cerebral ischemia is apparently rare in cases of spinal SAH, but we hypothesize that the large blood load in our patient increased the risk of the long-term consequences of SAH.

## 4. Conclusions

Our case description and examination of the pertinent literature yield the following salient points: (1) spontaneous spinal SAH can result in the same long-term sequelae typically associated with aneurysmal SAH, and the clinician must have a degree of suspicion in such patients; (2) the pathophysiological mechanisms underlying cerebral vasospasm may explain this clinical phenomenon; and (3) further elucidation of these mechanisms may provide better treatments for patients with SAH.
